# Retraction: The structure of SV40 large T hexameric helicase in complex with AT-rich origin DNA

**DOI:** 10.7554/eLife.46910

**Published:** 2019-04-03

**Authors:** Dahai Gai, Damian Wang, Shu-Xing Li, Xiaojiang S Chen

Gai D, Wang D, Li S-X, Chen XS. 2016. The structure of SV40 large T hexameric helicase in complex with AT-rich origin DNA. *eLife* **5**:e18129. doi: 10.7554/eLife.18129.

Published 6, December 2016

We are retracting the above *eLife* paper due to ambiguity of the density of the dsDNA, which is largely the result of the overlapping positioning of the dsDNA with the R3 crystallographic axis. Because of the 3-fold averaging in R3 symmetry and deformation of the bound dsDNA by the assembled helicase around it, the density of the DNA (especially the peripheral backbones) alone does not allow clear definition of the conformation of the DNA. As a result, while the structure of the protein itself largely holds true, we believe that it is appropriate to retract the paper because of the ambiguity of the DNA conformation as judged solely on the electron density. We thank Wim P Burmeister who originally brought the issue of R3 symmetry to our attention. All authors have agreed to the retraction and we will post an update if further information becomes available in the future.

